# Leveraging behavioral economics strategies to close gaps in biomedical HIV prevention

**DOI:** 10.1371/journal.pmed.1004475

**Published:** 2024-10-24

**Authors:** Jesse Heitner, Valerian Mwenda, Grace Umutesi, Ruanne V. Barnabas

**Affiliations:** 1 Division of Infectious Diseases, Massachusetts General Hospital, Boston, Massachusetts, United States of America; 2 National Cancer Control Program, Ministry of Health, Nairobi, Kenya; 3 Department of Global Health, University of Washington, Seattle, Washington, United States of America; 4 Department of Medicine, Harvard Medical School, Boston, Massachusetts, United States of America

## Abstract

Adolescent girls and young women (AGYW) in southern Africa face triple the HIV incidence of their male peers due to multiple factors, including economic deprivation and age-disparate relationships. A new study by Aurélia Lépine and colleagues has demonstrated that addressing healthcare costs among AGYW has the potential to reduce HIV incidence.

Globally, HIV incidence is falling due to tremendous advances in HIV treatment and prevention. However, gaps exist, with 1.3 million new infections globally in 2023 and the world remaining well behind on pre-exposure prophylaxis (PrEP) goals [1]. In 2023, the HIV incidence rate among adolescent girls and young women (AGYW) in southern Africa was over 3 times that of their male counterparts [1], with the gender disparity in Cameroon being a factor of 5 [2]. A known driver of this gender disparity is the economic marginalization of young women in sub-Saharan Africa; in this setting, transactional, age-disparate, and multiple concurrent sexual relationships can be a necessary means of economic survival [3]. Therefore, novel strategies are needed to protect key populations, including women engaging in transactional sex and commercial sex workers, who are 9 times more likely to acquire HIV than women in the general population [1].

Responding to this heightened vulnerability, Aurélia Lépine and colleagues are to be applauded for implementing the POWER study—a stratified randomized controlled trial that sought to empower women currently engaged in transactional or commercial sex in Yaoundé, Cameroon to have greater agency in their sexual health by providing them with health insurance. Financial supports for HIV prevention in the form of cash transfer programs, savings programs, and school cost reduction programs have been studied with mixed results [4], but to our knowledge, providing health insurance is a novel experimental strategy. The authors hypothesized that the intervention could affect HIV infection by increasing healthcare use for sexually transmitted infections (STIs) treatment, thus lowering HIV susceptibility or reducing a very common economic shock and thus reducing the need to engage in risky transactional or commercial sexual behaviors. The analysis compared randomized recipients and non-recipients, and stratified by engagement in either commercial or transactional sex—the former distinguished as female sex workers (FSW) and the latter distinguished as women leveraging “non-commercial sexual relationships in exchange for material support and benefits.” The 2 groups are often conflated, but as the authors note, they can have substantially different financial reliance on sex as well as outreach from and access to HIV prevention services.

Previous research on financial support for HIV prevention in other settings, primarily undertaken within Africa [4], has resulted in promising but mixed findings that are often stronger for the social determinants of health than HIV itself. In South Africa, Pettifor and colleagues assessed cash transfers conditional on school attendance among AGYW (HPTN 068) [5] and found that they did not significantly reduce HIV incidence, but recipients were significantly less likely to report experiencing partner physical violence, having a sexual partner, or engaging in unprotected sex. In Tanzania, a cash transfer program for AGYW aged 15 to 23 years not enrolled in school reduced their dependence on male sex partners who previously provided basic needs (such as food and toiletries) dependent on transactional sex [6]. Similar findings were reported in Malawi, Lesotho, and Eswatini; however, findings on efficacy to prevent HIV and STIs were mixed [4].

The intervention by Lépine and colleagues resulted in a significant reduction of the odds of acquiring HIV for the transactional sex strata (odds ratio = 0.109 [0.014, 0.870], adjusted OR = 0.113 [0.014, 0.912]). No impact on HIV incidence was measured among women engaging in commercial sex, and uneven, nonsignificant effects were reported on other STIs across strata. The authors further reported a significant increase in the likelihood of quitting transactional sex (25.5% versus 16.9%) as well as suggestive evidence of increased condom use among recipients in the transactional sex strata. Among commercial sex workers, the intervention did not change whether participants left commercial sex work nor used condoms. Recipients in both strata experienced greater protection from health-related economic shocks. Finding that the pattern of STI incidence did not support the hypothesized mechanism of increased STI care-seeking for reducing HIV incidence, the authors concluded that improved financial security was the likely mechanism reducing HIV incidence in the transactional sex stratum. These findings add to evidence that financial interventions, when well-tailored to the context, can empower women at risk to reduce their HIV exposure.

The innovative approach of providing health insurance rather than direct cash is compelling because health insurance could flexibly mitigate a key source of economic shocks as needed. Providing a flat rate in a cash transfer has the potential to be insufficient either to protect from larger shocks or be motivating to recipients, who often feel that the amount given is too little in relation to their total needs [7]. While experiments which randomly allocate health insurance are not entirely unique, investigations of their effects on health, as opposed to financial protection or healthcare utilization, are almost absent in low-income settings [8], and expanded research on the effects of its provision on health is warranted. The heterogeneous effects by strata are also compelling for future design considerations. Subgroup analyses of cash transfer programs often indicate that their benefits can vary considerably by individual characteristics such as sex, socioeconomic status, region, and education [9], and the same may well hold true for the effects of health insurance. “Know your epidemic” is a mantra that yet again applies.

Based on the findings, 3 areas of future research seem warranted. First, replication studies could better confirm financial protection as the HIV reducing mechanism if they included an objective measure of economic insecurity that was found to be an effect modifier of insurance provision. Metrics of economic insecurity and vulnerability abound, and locally appropriate ones should be selected and tailored to study context. Mediation analysis should also show differential HIV incidence between women who do and do not exit transactional sex. Second, health insurance can reasonably be expected to have health benefits beyond HIV, and exploring the full breadth of recipient benefits is warranted. Third, an intervention can be effective without being cost-effective, and the cost-effectiveness of health insurance should be estimated against other available HIV prevention interventions such as PrEP provision.

The fact that the research from Lépine and colleagues indicated intervention efficacy among women engaged in informal transactional sex and not commercial sex workers points to the wide breadth of an at-risk but protectable population and the consideration of household poverty in HIV prevention initiatives. Broad-based financial protection and poverty alleviation programs could be a practical strategy for AGYW, with additional strategies needed for commercial sex workers such as condom use promotion [10] or PrEP outreach efforts given its high acceptability [11]. Other interventions shown to reduce HIV incidence among AGYW, such as ensuring that girls attend and complete school, could be explored as excellent complimentary opportunities for investment. Policymakers should ensure broad stakeholder engagement and alignment before launching poverty alleviation programs. This would promote contextual tailoring, ensure buy-in, and optimize opportunities for sustainability, especially since such programs would ultimately need to be supported through local funding streams. Since evidence points to larger mortality-reducing effects for cash transfer programs that cover bigger segments of the population [12], it is key for stakeholders to map the specific needs of various groups to reach as many people as possible, and leverage interventions appropriate for each group. Combining interventions could be sustainable and efficacious; for instance, health insurance for AGYW with lower economic means combined with free health supplies such as sanitary pads or other supports to keep adolescent girls in schools. The ultimate aim of well-designed and tested economic interventions is to reduce economic inequalities and their impact on health outcomes.
